# Structural properties of thin-film ferromagnetic topological insulators

**DOI:** 10.1038/s41598-017-12237-2

**Published:** 2017-09-21

**Authors:** C. L. Richardson, J. M. Devine-Stoneman, G. Divitini, M. E. Vickers, C.-Z. Chang, M. Amado, J. S. Moodera, J. W. A. Robinson

**Affiliations:** 1 University of Cambridge, Department of Materials Science and Metallurgy, Cambridge, CB3 0FS United Kingdom; 2 Massachusetts Institute of Technology, Francis Bitter National Magnet Laboratory, Cambridge, MA 02139 USA; 3 Pennsylvania State University, Department of Physics, State College, PA, 16802-6300 USA; 4 Department of Physics, Massachusetts Institute of Technology, Cambridge, MA 02139 USA

## Abstract

We present a comprehensive study of the crystal structure of the thin-film, ferromagnetic topological insulator (Bi, Sb)_2−*x*_V_*x*_Te_3_. The dissipationless quantum anomalous Hall edge states it manifests are of particular interest for spintronics, as a natural spin filter or pure spin source, and as qubits for topological quantum computing. For ranges typically used in experiments, we investigate the effect of doping, substrate choice and film thickness on the (Bi, Sb)_2_Te_3_ unit cell using high-resolution X-ray diffractometry. Scanning transmission electron microscopy and energy-dispersive X-ray spectroscopy measurements provide local structural and interfacial information. We find that the unit cell is unaffected in-plane by vanadium doping changes, and remains unchanged over a thickness range of 4–10 quintuple layers (1 QL ≈ 1 nm). The in-plane lattice parameter (*a*) also remains the same in films grown on different substrate materials. However, out-of-plane the *c*-axis increases with the doping level and thicknesses >10 QL, and is potentially reduced in films grown on Si (1 1 1).

## Introduction

The quantum anomalous Hall effect (QAHE) allows the resistance quantization and dissipationless edge states seen in the quantum Hall effect^1^, but without the need for an applied magnetic field. The topologically-protected edge states of the QAHE are also chiral and spin-polarised^2,3^, acting as a natural spin filter. Additional applications are starting to be explored, for example in spintronics as a pure spin current source or detector^4^, and in topological quantum computing^5,6^.

In experimental devices, the QAHE is remarkably robust, and has been observed in chromium- and vanadium-doped (Bi, Sb)_2_Te_3_ across a range of film thicknesses, and grown on multiple substrates^7–11^. Quantization has been improved by reducing film thickness and doping with vanadium to suppress dissipative channels^12^, but whether these variables affect the crystal structure and electronic band structure has largely been overlooked experimentally. To date, the *a*-axis parameter has only been determined for Cr-doped (Bi, Sb)_2_Te_3_ grown on SrTiO_3_ (1 1 1) with or without a Te capping layer^13^, with no systematic study of the role of thickness or doping level. This is despite the potential for uniaxial or biaxial strain in the films to either drive these materials into the topologically trivial (non-QAH) regime by altering the band structure^14^, or affect the fabrication and performance of ferromagnetic/non-ferromagnetic topological insulator heterostructure devices due to lattice mismatch^15–17^.

Here, we conduct a comprehensive study of the effect of thickness, vanadium-doping and substrate choice on the crystal structure of MBE-grown (Bi, Sb)_2−*x*_V_*x*_Te_3_ thin films, and compare to existing results in the literature. We use high-resolution X-ray diffractometry (HRXRD) to determine the in- and out-of-plane lattice parameters, orientation relationships and epitaxial quality of the film, substrate, and Te capping layer. The HRXRD data is supported by information about the local structure and doping, provided by scanning transmission electron microscopy (STEM) and energy-dispersive X-ray spectroscopy (EDX).

## Results

### Crystallinity and elemental composition

**Figure 1:**
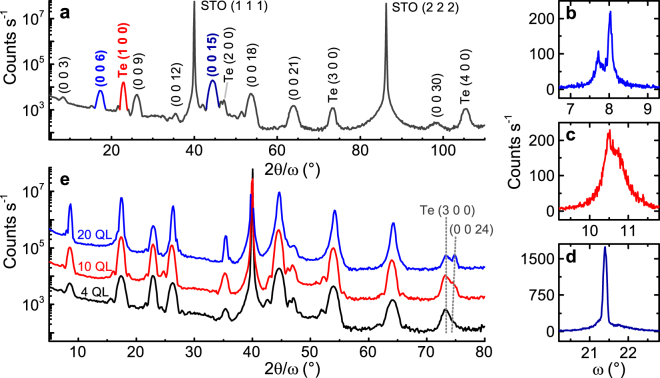
High-resolution X-ray diffractometry of (Bi, Sb)_2−*x*_V_*x*_Te_3_ thin films on SrTiO_3_ (1 1 1) with 10 nm tellurium capping layer. (**a**) Indexed 2*θ*/*ω* scan of a 4 QL film (quintuple layer, 1 QL ≈ 1 nm; x = 0.11). (Bi, Sb)_2−*x*_V_*x*_Te_3_ peaks are unnamed. (**b**–**d**) Rocking curves of (0 0 6), Te (1 0 0) and (0 0 15) peaks (shown in (**a**) in blue, red and dark blue, respectively). (**e**) 2*θ*/*ω* scans of 4 (black), 10 (red) and 20 QL (blue) films (x = 0.06–0.07, 10 and 20 QL data offset for clarity). Grey dashed lines are guides to the eye, showing the contraction of the lattice as thickness increases.

We first demonstrate the quality of the films with symmetrical high-resolution X-ray diffraction measurements, probing planes parallel to the substrate. Figure 1a shows a 2*θ*/*ω* scan of a 4 QL (quintuple layer, each QL ≈ 1 nm) film of (Bi, Sb)_1.89_V_0.11_Te_3_ on a SrTiO_3_ (1 1 1) substrate, with a 10 nm Te capping layer. We observe (Bi, Sb)_2−*x*_V_*x*_Te_3_ peaks at (0 0 3*n*), as expected for its space group $R\bar{3}m$, and tellurium peaks at (*m* 0 0), echoing previous results on Cr-doped (Bi, Sb)_2_Te_3_^12,13^.

Both layers are highly-oriented and low in defects, as shown by the rocking curves in Fig. 1b–d (rocking curves taken on the (Bi, Sb)_2−*x*_V_*x*_Te_3_ (0 0 6), Te (1 0 0) and (Bi, Sb)_2−*x*_V_*x*_Te_3_ (0 0 15) peaks, respectively). The (Bi, Sb)_1.89_V_0.11_Te_3_ (Fig. 1b,d) has a smaller full-width at half-maximum (FWHM, Gaussian fit) than tellurium: ~0.11° as opposed to 0.65°. The (Bi, Sb)_2−*x*_V_*x*_Te_3_ (0 0 6) curve shows dual peaks ~0.15° from the expected value of *ω*, consistent with the observation of twinned crystal domains in Cr-doped films^18^.

These results hold for all samples grown on SrTiO_3_ (1 1 1), even with changes to doping and thickness. Figure 1e shows 2*θ*/*ω* scans of samples with *x* = 0.06–0.07 and thicknesses of 4, 10 and 20 QLs (black, red and blue, respectively). The plots are offset for clarity. The changes in doping and thickness do not affect the relative intensities of the peaks. The (Bi, Sb)_2−*x*_V_*x*_Te_3_ *c*-axis appears to shorten as the thickness increases; this is clearly seen in the 2*θ* position of the (0 0 24) peak as it changes relative to the Te (3 0 0) peak, which in turn stays constant with respect to SrTiO_3_ (1 1 1). However, averages of Gaussian fits to 3 reflections show that 4 and 10 QL films have approximately equal *c* (30.51 ± 0.05 Å and 30.54 ± 0.04 Å respectively, compared to *c* = 30.44 ± 0.02 Å for the 20 QL film). The 3 reflections chosen (*l* = 15, 18 and 21) were unaffected by a possible sample displacement error.

HRXRD measurements of 10 QL films grown on Al_2_O_3_ (0 0 0 1) and Si (1 1 1) show very similar epitaxial growth and out-of-plane lattice parameters (See Supplementary Fig. S1). The (Bi, Sb)_2−*x*_V_*x*_Te_3_ lattice constant *c* = 30.47 ± 0.08 Å and 30.45 ± 0.07 Å, respectively, a slight decrease compared to 30.54 ± 0.03 Å on SrTiO_3_ (1 1 1) (10 QL film). On an Al_2_O_3_ (0 0 0 1) substrate, rocking curves on (Bi, Sb)_2−*x*_V_*x*_Te_3_ (0 0 15) and Te (1 0 0) have a FWHM of 0.12° and 0.77°, respectively, similar to the SrTiO_3_ (1 1 1) samples. Growth appears to be more disordered on Si (1 1 1), where the (Bi, Sb)_2−*x*_V_*x*_Te_3_ (0 0 15) rocking curve has a larger FWHM of ~0.36°.

**Figure 2:**
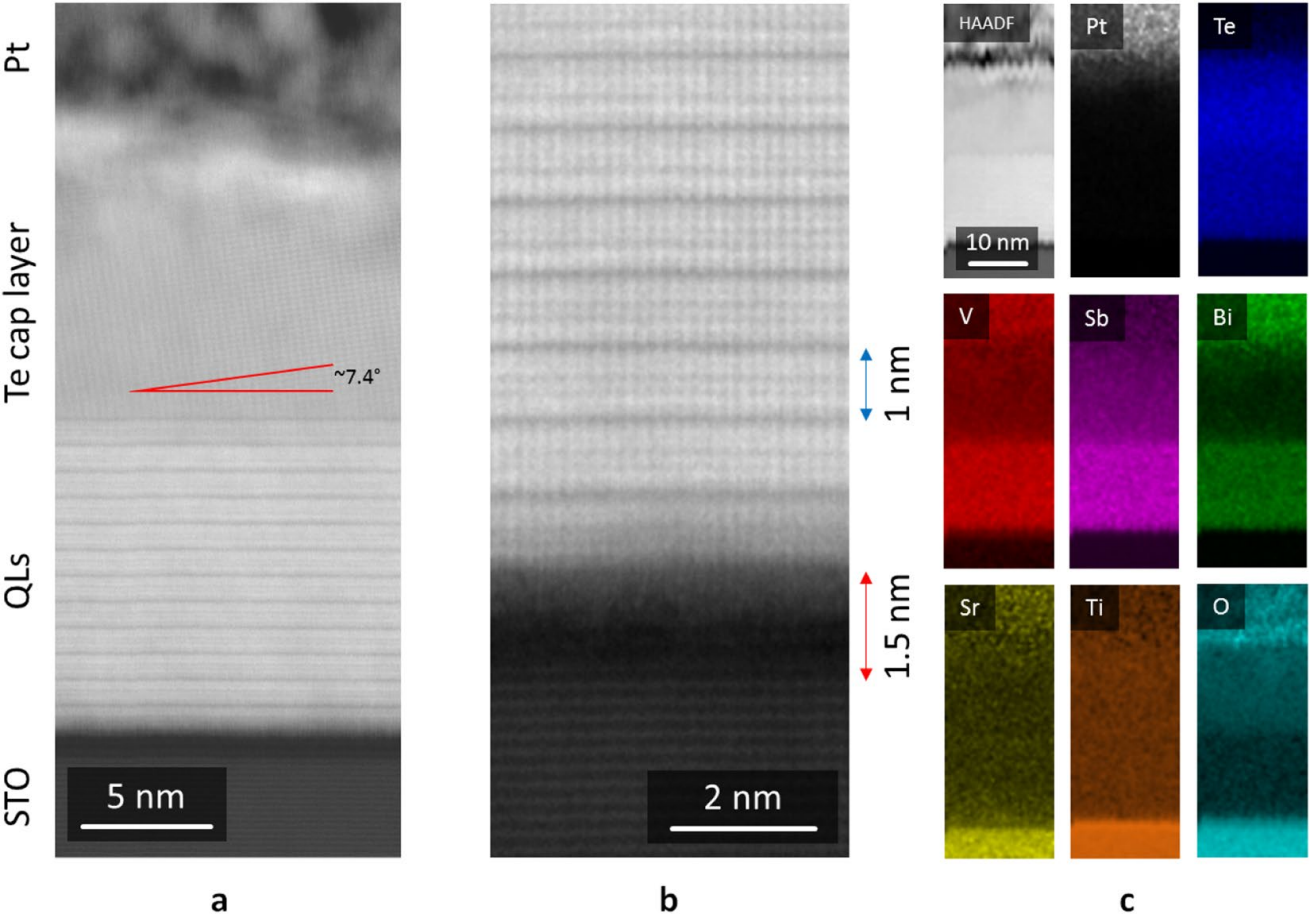
Scanning transmission electron microscopy (STEM) and elemental analysis of (Bi, Sb)_2−*x*_V_*x*_Te_3_ on SrTiO_3_ (x = 0.07). (**a**) STEM cross-sectional view of the film. (**b**) HRSTEM view of the substrate-film interface. (**c**) HAADF reference image and EDX elemental maps.

We now investigate the local crystallinity and interfaces using scanning transmission electron microscopy (STEM). The High-Angle Annular Dark-Field (HAADF) signal, generating the images shown in Fig. 2, is proportional to the local thickness and atomic number. For a relatively homogeneous thickness, such as a TEM lamella, the brightness is proportional to the average Z for a given pixel such that brighter atomic columns correspond to heavier atoms.

**Figure 3:**
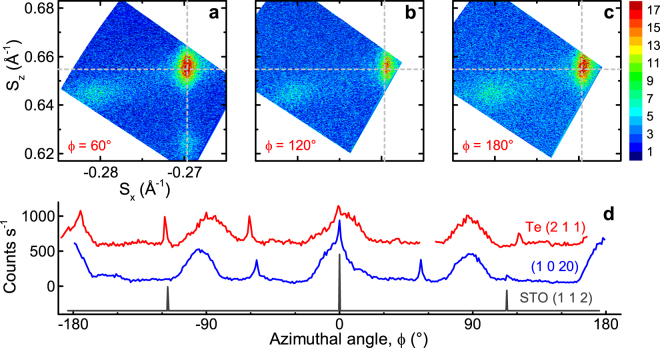
In-plane orientation of layers. (**a**–**c**) Reciprocal space maps of the (1 0 20) and Te (2 1 1) peaks of a 10 QL, x = 0.07 film, at azimuthal angles *ϕ* = 60°, 120° and 180° respectively. Grey dashed lines indicate fitted peak value from data in Fig. 5b. Film peaks measured with respect to SrTiO_3_ (1 1 2). (**d**) Azimuthal angle scans of substrate (grey, STO (1 1 2): 2*θ* = 57.84°, offset = 19.50°), topological insulator (blue, (1 0 20): 2*θ* = 66.10°, offset = 23.34°) and capping layer (red, Te (2 1 1): 2*θ* = 65.56°, offset = 24.78°) peaks, offset for clarity. The substrate and topological insulator peaks have the expected 3- and 6-fold symmetries, whereas Te (2 1 1) has 6-fold rather than 2-fold symmetry.

We observe regular growth of the QLs, with homogeneous thickness of ~1 nm for each layer (Fig. 2a,b). The Te capping layer is crystalline and appears to grow at an angle of ~7° to the underlying quintuple layers. However, detailed HRXRD indicates that this is not the case (see Fig. 3 and discussion). While above the first QL we observe good epitaxial growth, the interface between STO and the QLs is relatively irregular, with the first QL being discontinuous and heavy atomic species coexisting with the lighter substrate. The SrTiO_3_ substrate also displays a change in contrast over a thickness of ~1.5 nm at the substrate surface, where a lower HAADF signal suggests that only light elements are present.

STEM-EDX (energy-dispersive X-ray spectroscopy) elemental maps are reported in Fig. 2c (non-negative matrix factorisation (NMF) of the EDX data is shown in Supplementary Fig. S2). The Pt signal originates from the protective layer deposited during focused ion beam (FIB) sample preparation. The (Bi, Sb)_2−*x*_V_*x*_Te_3_ region contains strong signals from Bi, Sb, V and Te, which are all homogeneous throughout the film thickness. Tellurium extends above the (Bi, Sb)_2−*x*_V_*x*_Te_3_ into the capping layer. As expected, Sr, Ti and O signals dominate the substrate region. However, Ti and O extend further towards the (Bi, Sb)_2−*x*_V_*x*_Te_3_ compared to Sr, indicating that the interfacial region that appears darker in the STEM-HAADF images has a lower Sr concentration than the bulk SrTiO_3_.

Having confirmed that the films are epitaxial and homogeneously doped, we use reciprocal space mapping of asymmetrical peaks (those whose corresponding atomic planes are not parallel to the substrate) to find the in-plane orientations of the substrate, ferromagnetic topological insulator, and capping layer. Figure 3a–c shows reciprocal space maps of a 10 QL, x = 0.07 sample, where *S*_*x*_ is the reciprocal of the in-plane d-spacing, and *S*_*z*_ is the reciprocal of the out-of-plane d-spacing. The three maps show equivalent areas of reciprocal space at three different azimuthal angles. The maps include the (Bi, Sb)_2−*x*_V_*x*_Te_3_ (1 0 20) and Te (2 1 1) peaks (right- and left- hand side of the panels, respectively). Figure 3a also shows (Bi, Sb)_2−*x*_V_*x*_Te_3_ (1 0 19) directly below (1 0 20).

Whilst the higher-intensity (1 0 20) peak has the expected six-fold symmetry of a (Bi, Sb)_2_Te_3_-based compound, the appearance of the Te (2 1 1) peak at all three angles indicates that the capping layer grows epitaxially in three equivalent orientations. Azimuthal angle scans confirm these findings (Fig. 3d), and indicate the epitaxial relationship (Bi, Sb)_2−*x*_V_*x*_Te_3_ [0 1 0]//SrTiO_3_ [1 1 $\bar{2}$]. Note that one of the Te peaks was not captured in this scan due to slight sample misalignment, and that the broader peaks visible on the thin-film scans are due to sample geometry, confirmed by repeating the measurement away from the peaks. We also observe a Te (2 1 0) peak at Δ*ϕ* ≈ 53° from Te (2 1 1), confirming that the *a*- and *c*-axes of the tellurium cap are in-plane (see Supplementary Fig. S3), and an epitaxial relationship of (Bi, Sb)_2−*x*_V_*x*_Te_3_ [1 0 0]//Te [$\bar{1}$ 2 2]. Since the crystallographic axes are in-plane, we conclude that the ~7.4° tilt observed with STEM is not a tilt of the unit cell itself, but rather an alignment of Te atoms revealed by an off-axis cut.

### Thickness, doping and substrate dependence of lattice parameters

**Figure 4:**
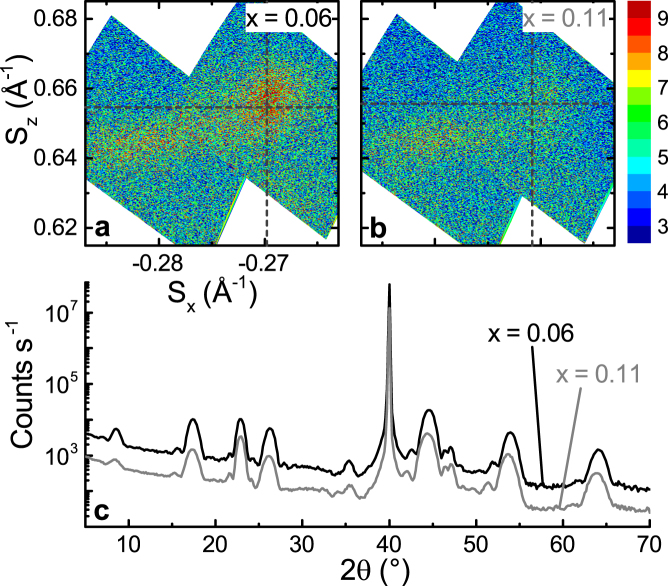
Effect of vanadium doping on the unit cell. Reciprocal space maps of the (1 0 20) and Te (2 1 1) (right and left peaks in panels, respectively) in films on SrTiO_3_, with (**a**) x = 0.06 and (**b**) x = 0.11, where (Bi, Sb)_2−*x*_V_*x*_Te_3_. Dashed lines indicate fitted position of (1 0 20). (**c**) 2*θ*/*ω* scans (offset for clarity) of the same films.

**Figure 5:**
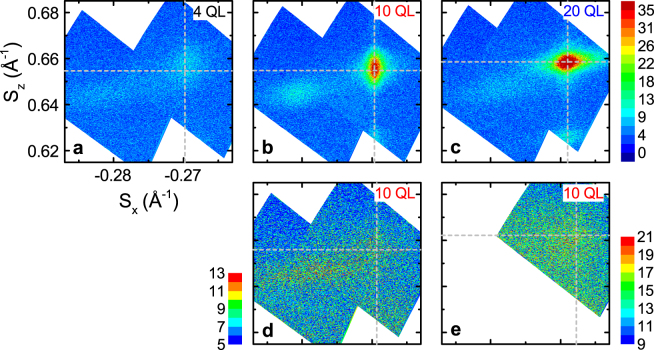
Unit cell parameters as a function of thickness and substrate choice. Reciprocal space maps showing the (Bi, Sb)_2−*x*_V_*x*_Te_3_ (1 0 20) and Te (2 1 1) peaks, from films grown on SrTiO_3_ (1 1 1) ((**a**–**c**) 4, 10 and 20 QL, respectively), Al_2_O_3_ (0 0 0 1) ((**d**) 10 QL), and Si (1 1 1) ((**e**) 10 QL). Film peaks measured with respect to SrTiO_3_ (1 1 2), Al_3_O_3_ (0 1–1 8) or Si (3 3 1). Grey dashed lines indicate fitted position of (1 0 20). Panel (a) shows the same dataset as Fig. 4a.

To determine whether the unit cell of (Bi, Sb)_2−*x*_V_*x*_Te_3_ changes with doping level or film thickness, we repeat the reciprocal space measurements detailed above. A longer counting time is used in order to precisely determine the in- and out-of-plane lattice parameters. Figure 4 is a comparison of 4 QL films with *x* = 0.06 and 0.11, and Fig. 5 shows reciprocal space maps for 4, 10 and 20 QL films on SrTiO_3_, and 10 QL films on Al_2_O_3_ (0 0 0 1) and Si (1 1 1). Table 1 summarises the calculated lattice parameters, along with previous HRXRD results from the literature for comparison.

**Table 1:** Unit cell parameters of Cr- and V-doped (Bi, Sb)_2_Te_3_ calculated from HRXRD measurements of samples with various substrates, thicknesses and doping levels.

Substrate	Thickness	Doping	c (Å, 2*θ*/*ω*)	c (Å, RSM)	a (Å, RSM)	V (Å^3^)	Lattice mismatch	FWHM (°)
Bulk				30.60	4.30	490.0		
STO (1 1 1)	4 QL	V_0.06_	30.51 ± 0.05	30.55 ± 0.42	4.28 ± 0.14	484.0	−3.4% $(\frac{2{a}^{TI}}{3}\,\cos \,30^\circ )$	
		V_0.11_	30.62 ± 0.06	30.50 ± 0.42	4.26 ± 0.15	481.2		0.11
	5 QL^a^	Cr_0.15_*	30.62 ± 0.06		4.28	485.8		
		Cr _0.15_	30.66 ± 0.19		4.28	486.4		
	10 QL	V_0.07_	30.54 ± 0.03	30.54 ± 0.43	4.28 ± 0.16	484.5		0.15
	20 QL	V_0.07_	30.44 ± 0.01	30.34 ± 0.41	4.30 ± 0.16	487.4		0.16
Al_2_O_3_ (0 0 0 1)	10 QL	V_0.07_	30.47 ± 0.08	30.49 ± 0.32	4.29 ± 0.13	485.6	+2.6% $(\frac{2{a}^{TI}}{3})$	0.12
	20 QL^b^	V_0.15_	30.39					
		Cr_0.16_	30.15					
		None	30.34					
Si (1 1 1)	10 QL	V_0.07_	30.45 ± 0.07	30.22 ± 0.41	4.30 ± 0.20	487.6	−2.8% $(\frac{3{a}^{TI}}{4})$	0.36
InP (1 1 1)	8 QL^c^	Cr_0.22_	30.26				−3.6% (*a* ^*TI*^ cos 30°)	

Parameters from this study are calculated from three (0 0 *n*) reflections (column labeled 2*θ*/*ω*) or a single (1 0 20) reflection (columns labeled RSM, from reciprocal space maps). Also included are unit cell volume (column labelled V, calculated from columns *c* (2*θ*/*ω*) and *a* (RMS)), lattice mismatch with respect to each substrate, and the full-width at half-maximum (FWHM) of rocking curves taken on the (0 0 15) peak.

*Uncapped.

^a^J. Park *et al*.^13^

^b^C.-Z. Chang *et al*.^12^.

^c^J. Checkelsky *et al*.^8^.

Reciprocal space maps (Fig. 4a and b, x = 0.06 and 0.11, respectively) and 2*θ*/*ω* scans (Fig. 4c, *x* = 0.11 data (grey) offset for clarity) indicate that the *c*-axis lengthens as the doping is increased. From the asymmetrical peaks (Fig. 4a,b), *c* = 30.55 ± 0.42 Å for *x* = 0.06 and 30.50 ± 0.42 Å for *x* = 0.11, whereas from the symmetrical peak measurements (Fig. 4c), *c* = 30.51 ± 0.05 Å and 30.62 ± 0.06 Å. For both doping levels, *c* is still close to the bulk value of 30.60 Å.

The in-plane lattice parameter is also close to the bulk value (*a* = 4.30 Å). For the *x* = 0.06 and 0.11 films, the *a*-axis parameter is 4.28 ± 0.14 Å or 4.26 ± 0.15 Å, respectively, and matches to the effective in-plane spacing of the substrate (3.565 Å) with a 30° rotation (*a* ^*TI*^ cos 30° = 3.71 Å). The slight discrepancy may be due to the Sr-deficient interfacial layer shown in Fig. 2, which appears to have a larger lattice than the bulk of the substrate.

The in-plane parameter also remains constant as a function of thickness (Fig. 5a–c): the 10 and 20 QL samples (*x* = 0.07, Fig. 5b,c) have *a* = 4.28 ± 0.16 Å and 4.30 ± 0.16 Å. Out-of-plane, the thickest (20 QL) film has a smaller *c*, at 30.34 ± 0.41 Å. The 10 and 4 QL films have similar unit cells, with *c* = 30.54 ± 0.43 Å and 30.55 ± 0.42 Å.

Figure 5b,d,e shows data taken for 10 QL films grown on SrTiO_3_ (1 1 1), Al_2_O_3_ (0 0 0 1) and Si (1 1 1), respectively. There is very little difference between the in-plane parameters on the three substrates: *a* = 4.28 ± 0.16, 4.29 ± 0.04 and 4.30 ± 0.20 Å, respectively. Out-of-plane, the film grown on Si (1 1 1) appears to have a shorter unit cell, 30.22 ± 0.41 Å as opposed to 30.49 ± 0.32 Å on Al_2_O_3_ and 30.54 ± 0.43 Å on SrTiO_3_. The Te (2 1 1) peak was not observed for the Si (1 1 1) sample; based on the relative peak breadths and intensities of symmetric 2*θ*/*ω* scans, the Te capping layer was too thin (2–5 nm) for the peak to be detected (see Supplementary Fig. S1). The peaks measured on Al_2_O_3_ (0 0 0 1) and Si (1 1 1) were all less intense and broader than for the equivalent film grown on SrTiO_3_ (1 1 1), which indicates less well-defined crystallographic orientation in-plane. Whilst the thickness of the films contributes to the breadth and low intensity of the peaks, Scherrer fits to the symmetrical data show that the nominally 10 QL films on SrTiO_3_ (1 1 1), Al_2_O_3_ (0 0 0 1) and Si (1 1 1) are 9.9 ± 1.9 nm, 10.8 ± 0.1 nm and 7.2 ± 0.8 nm, respectively, which does not correlate to the observed difference in intensity.

## Discussion

**Figure 6:**
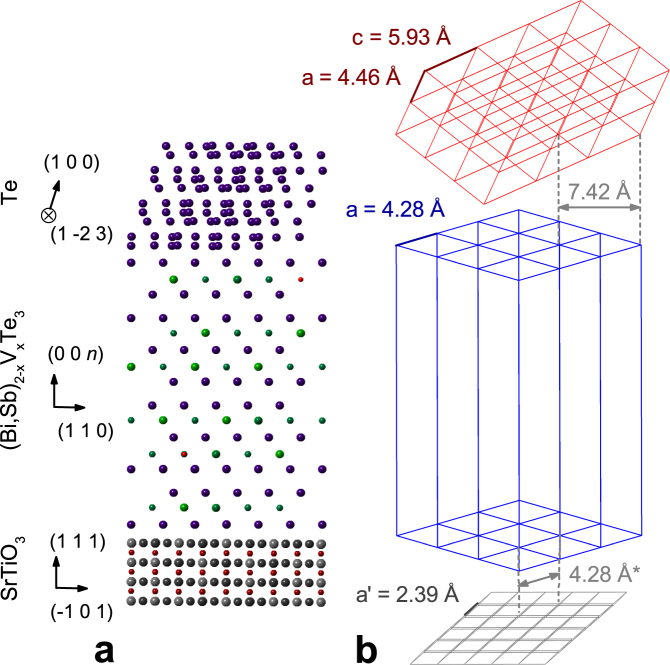
Schematic of SrTiO_3_/(Bi, Sb)_2−*x*_V_*x*_Te_3_/Te. (**a**) View along the (Bi, Sb)_2−*x*_V_*x*_Te_3_ ($\bar{1}$ 1 0) direction (Te atoms = dark blue, Sb = dark green, Bi = light green, V = red, Sr = grey, Ti = dark red, O = dark grey). Parameters used are those of the 10 QL film. (**b**) Unit cells and experimental lattice parameters of the substrate and films, demonstrating in-plane lattice matching.

Our results, and those from previous HRXRD studies of ferromagnetic (Bi, Sb)_2_Te_3_, are presented in Table 1. Figure 6 is a schematic summarizing the results for 10 QL of (Bi, Sb)_2−*x*_V_*x*_Te_3_ on SrTiO_3_, with a Te capping layer. Surprisingly, measurements of the in-plane lattice constant of these quantum anomalous Hall insulators have only been made for Cr-doped films grown on SrTiO_3_ (see Table 1), even though the QAHE has also been observed in (Bi, Sb)_2−*x*_Cr_*x*_Te_3_ on InP (1 1 1)^8,19^, Si (1 1 1)^9,20^ and GaAs (1 1 1)^10,21^, and grown on Al_2_O_3_ (0 0 0 1)^12^. Vanadium-doped films have not previously been characterised in-plane.

Although we do not observe large differences in the (Bi, Sb)_2−*x*_V_*x*_Te_3_ unit cell between various doping levels, thicknesses or substrates, there are small trends in *c*, whereas *a* remains unchanged across all the samples measured. The latter indicates that (Bi, Sb)_2_Te_3_ does not strongly match to the substrate, perhaps due to weak, Van der Waals bonding between quintuple layers and the substrate. Doping with vanadium or chromium slightly lengthens the unit cell along the *c*-axis, which we observe in our more accurate symmetrical measurements, though asymmetrical measurements show no difference between our V-doped samples since asymmetrical peaks are much broader.

Where data is available for comparison, 20 QL films have a shorter unit cell than 4–10 QL films. This could be due to changing conditions during growth, or perhaps a gradient in the doping after around 10 QL (up to at least 10 QL, V-doping is homogeneous, as shown by EDX). Finally, the unit cells of films grown on Al_2_O_3_ (0 0 0 1) and SrTiO_3_ (1 1 1) are almost identical, but the average value of *c* on Si (1 1 1) is smaller. This is not a statistically significant trend (see errors quoted in Table 1), but it is consistent across all our measurements. This may correlate with the greater disorder in this sample (demonstrated by a wider rocking curve–see Supplementary Fig. S1), which indicates that Si (1 1 1) is a less suitable substrate for (Bi, Sb)_2−*x*_V_*x*_Te_3_. However, this does not seem to be due to lattice mismatch, which is roughly the same as for SrTiO_3_ (1 1 1) and Al_2_O_3_ (0 0 0 1) (see Table 1).

Although the small changes in the unit cell within the parameters used here indicate that strain engineering is not feasible, the lack of biaxial strain in particular is important for growth of doped/undoped heterostructures such as those in ref.^17^.

We also find that the crystal structure of the V-doped films is unchanged compared to Cr-doped (Bi, Sb)_2_Te_3_^13^, and that the Te capping layer is epitaxial and grows in three equivalent orientations on (Bi, Sb)_2_Te_3_. For all our samples, we observe the epitaxial relationship (Bi, Sb)_2_Te_3_ [1 0 0]//Te [$\bar{1}$ 2 2]. The slight out-of-plane disorder in the Te capping layer (observed as a larger FWHM of the rocking curve than (Bi, Sb)_2−*x*_V_*x*_Te_3_) is probably due to dislocations at monocrystalline domain boundaries or island formation on the top of the topological insulator during growth (as observed in STEM measurements).

In summary, we have investigated the crystal structure of the ferromagnetic topological insulator (Bi, Sb)_2−*x*_V_*x*_Te_3_ as a function of doping level, thickness and substrate, using high-resolution X-ray diffractometry supported by STEM and EDX. Focusing on the range commonly used in devices, we find that the unit cell is largely unaffected by vanadium doping changes, and remains unchanged over a thickness range of 4–10 quintuple layers (4–10 nm), showing only a slight positive trend as they increase. Substrate choice does not affect the in-plane lattice parameter (*a*), however, out-of-plane the *c*-axis appears to be weakly reduced in films grown on Si (1 1 1). These results are consistent with previous studies of ferromagnetic topological insulators. We also confirm the previous results of Park *et al*.^13^ regarding the Te capping layer growth and orientation, which grows epitaxially in three equivalent orientations on (Bi, Sb)_2_Te_3_. Since the in-plane lattice parameter remains constant over this experimentally-relevant range, heterostructures of doped and undoped (Bi, Sb)_2_Te_3_ (for example, REFs^16^,^17^) are a more promising route to applications than devices which rely on inducing strain to tailor the electronic band structure (as in REF.^14^).

## Methods

Vanadium-doped bismuth antimony telluride films were grown on SrTiO_3_ (1 1 1), Al_2_O_3_ (0 0 0 1) and Si (1 1 1) substrates by molecular beam epitaxy, and capped with 10 nm of tellurium (a detailed description is given in Chang *et al*.^12^). The Bi:Sb ratio in the films is 0.3:0.7 (except for the 10 and 20 QL films on SrTiO_3_ (1 1 1), which have 0.2:0.8) and is chosen such that the Fermi level is close to the Dirac point of the surface states.

We performed high-resolution X-ray diffractometry on 4, 10 and 20 quintuple-layer films in a Panalytical Empyrean (Series 2) *θ*-*θ* diffractometer. Optics were optimised for intensity over resolution due to the film thickness. We used a Ge(2 2 0) hybrid monochromator (for ${{\rm{Cu}}}_{{\kappa }_{\alpha }}$), and a 1/2° divergence slit on the incident beam. Diffracted-beam optics comprised either a Xe proportional counter and 1 mm beam tunnel (for symmetrical 2*θ*/*ω* measurements and rocking curves), or a PIXcel^3*D*^ (Medipix2) detector in 1D frame grab mode (for asymmetrical reciprocal space measurements). Typically, a step time of at least 10 s was required to detect the thin-film peaks, whereas the data shown in Fig. 5 has a step time of 20 minutes. Reciprocal-space maps are measured relative to the STO (1 1 2), Al_2_O_3_ (0 $\bar{1}$ 8) or Si (3 3 1) peak.

A cross-sectional sample for TEM analysis was prepared using a dual beam FIB/SEM (FEI Helios Nanolab), from a 10 quintuple-layer film on SrTiO_3_ (1 1 1). STEM-EDX analysis was carried out in a FEI Osiris, operated at 200 kV, equipped with a set of four EDX detectors in a cross configuration (Super-X by Bruker). Elemental maps were denoised using Principal Component Analysis (PCA) routines integrated in Hyperspy, an open source toolkit for EM data analysis, and the maps shown in Fig. 2c are 100 × 40 px, with a px size 0.5 nm × 0.5 nm and Gaussian blur 0.5 px for display. The High Resolution STEM images (HRSTEM) were acquired on a probe-corrected FEI Titan with an acceleration voltage of 300 kV.

### Data availability

The data presented in this manuscript is available at https://doi.org/10.17863/CAM.10453.

## Electronic supplementary material

Supplementary Information: Structural properties of thin-film ferromagnetic topological insulators
