# Supplementary material for: Structural properties of thin-film ferromagnetic topological insulators
Source: Sci Rep. 2017 Sep 21;7:12061. doi: 10.1038/s41598-017-12237-2 (PMC5608805; doi:10.1038/s41598-017-12237-2)
Supplement: Supplementary file 1 — Supplementary Information: Structural properties of thin-film ferromagnetic topological insulators [file 41598_2017_12237_MOESM1_ESM.pdf]

Supplementary Information: Structural properties of thin-film ferromagnetic topological insulators

C. L. Richardson[1], J. M. Devine-Stoneman[1], G. Divitini[1], M. E. Vickers[1], C.-Z. Chang[2,3], M. Amado[1], J. S. Moodera[2], and J. W. A. Robinson[1,*]

[1]University of Cambridge, Department of Materials Science and Metallurgy, Cambridge, CB3 0FS, UK
[2]Massachusetts Institute of Technology, Francis Bitter National Magnet Laboratory, Cambridge, MA 02139, USA
[3]Pennsylvania State University, Department of Physics, State College, PA 16802-6300, USA
[*]jjr33@cam.ac.uk

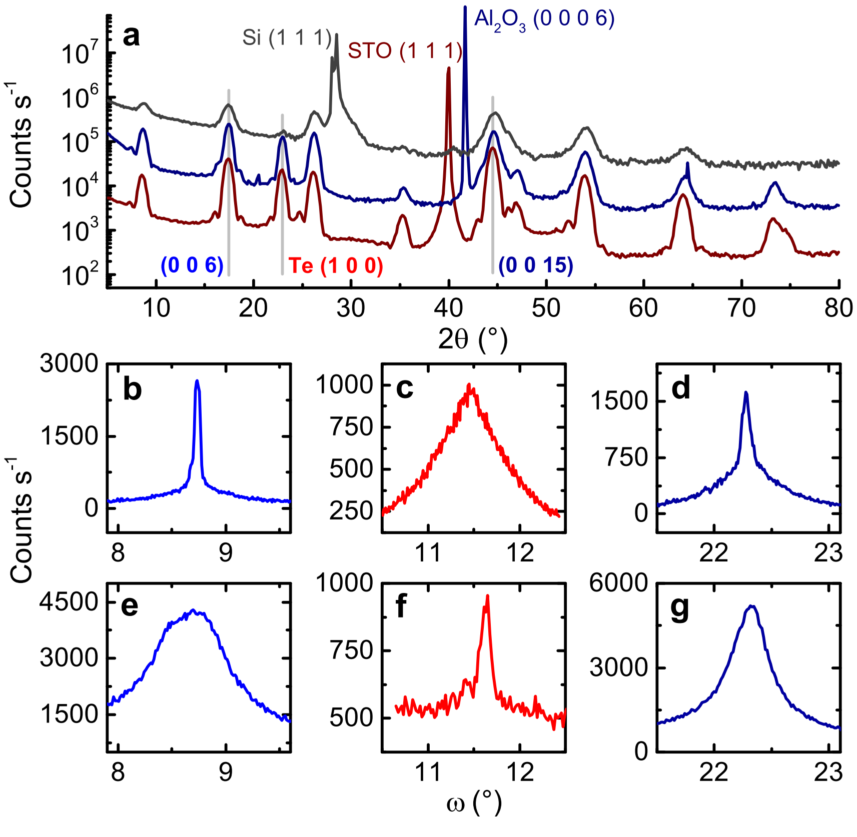

Figure S1. HRXRD measurements of 10 QL (Bi, Sb)$_{2-x}$V$_x$Te$_3$ films grown on SrTiO$_3$ (1 1 1), Al$_2$O$_3$ (0 0 0 1) and Si (1 1 1). **a** $2\theta/\omega$ scans of films grown on SrTiO$_3$ (1 1 1) (dark red), Al$_2$O$_3$ (0 0 0 1) (dark blue) and Si (1 1 1) (grey). The much lower intensity of the Te (1 0 0) peak on Si (1 1 1) indicates a capping layer thickness below 5 nm. **b-d** Rocking curves from the Al$_2$O$_3$ (0 0 0 1) sample, taken on the (Bi, Sb)$_{2-x}$V$_x$Te$_3$ (0 0 6), Te (1 0 0) and (Bi, Sb)$_{2-x}$V$_x$Te$_3$ (0 0 15) peaks, respectively. The rocking curve widths are very similar to those of the SrTiO$_3$ (1 1 1) sample. **e-g** Equivalent rocking curves from the Si (1 1 1) sample, with a larger width indicating more disordered growth of the (Bi, Sb)$_{2-x}$V$_x$Te$_3$ on this substrate.

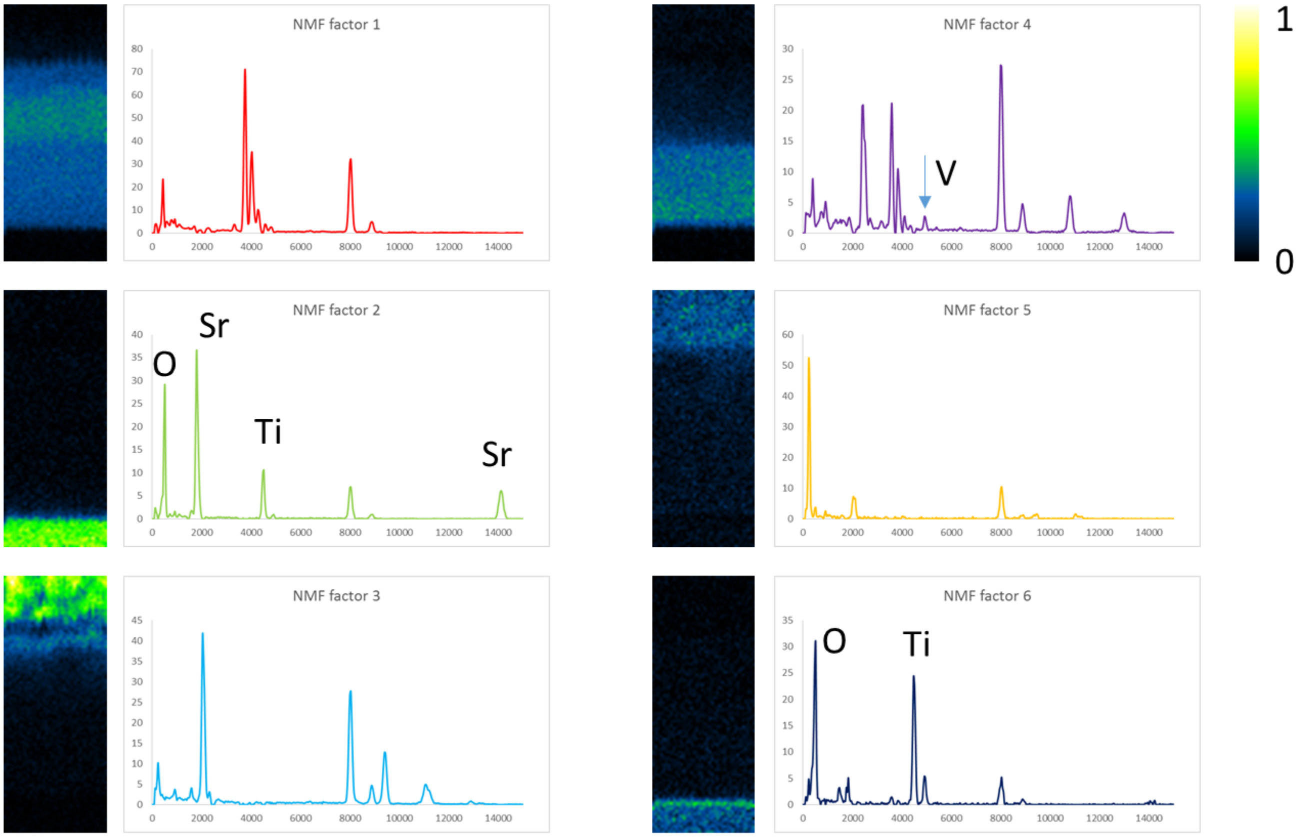

Figure S2. Non-negative matrix factorisation (NMF) of the EDX dataset, showing layer composition. Factor 4 (upper right) contains the QL region of the film, and has features corresponding to all the expected elements (Bi, Sb, Te, V). Factor 6 (lower right) represents a combination of Ti and O (with very little Sr) and is concentrated at the $SrTiO_3/(Bi, Sb)_{2-x}V_xTe_3$ interface.

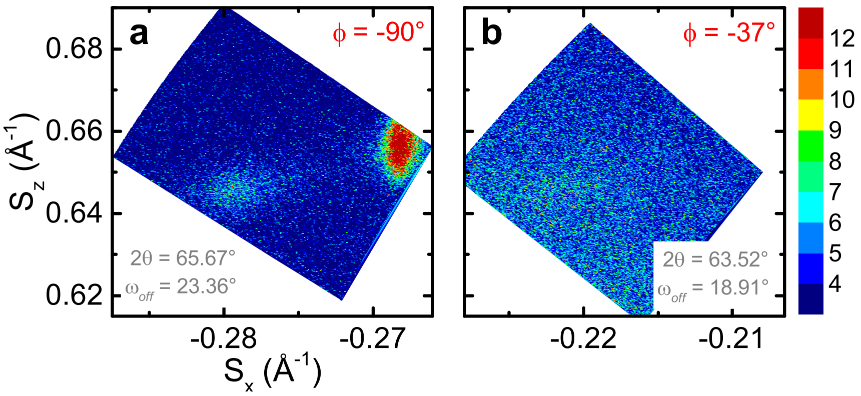

Figure S3. HRXRD confirming in-plane orientation of Te capping layer. **a** Reciprocal-space map showing Te (2 1 1) and $(Bi, Sb)_{2-x}V_xTe_3$ peaks at ϕ = -90°. **b** Te (2 1 0) peak measured at ϕ = -37°. $\Delta\phi \approx 53°$, matching the calculated difference in ϕ (53.05°) between the two peaks when Te (1 0 0) is normal to the substrate and confirming that the *a*- and *c*-axes are in the plane of the substrate.
